# FPGA Readout for Frequency-Multiplexed Array of Micromechanical Resonators for Sub-Terahertz Imaging

**DOI:** 10.3390/s24227276

**Published:** 2024-11-14

**Authors:** Leonardo Gregorat, Marco Cautero, Alessandro Pitanti, Leonardo Vicarelli, Monica La Mura, Alvise Bagolini, Rudi Sergo, Sergio Carrato, Giuseppe Cautero

**Affiliations:** 1 Dipartimento di Ingegneria e Architettura, Università degli Studi di Trieste, Piazzale Europa 1, 34127 Trieste, Italy; leonardo.gregorat@phd.units.it (L.G.); carrato@units.it (S.C.); 2 Elettra-Sincrotrone Trieste S.C.p.A. Science Park, Strada Statale 14, km 163.5, 34149 Basovizza, Italy; rudi.sergo@elettra.eu (R.S.); giuseppe.cautero@elettra.eu (G.C.); 3 Dipartimento di Fisica, Università degli Studi di Trieste, Piazzale Europa 1, 34127 Trieste, Italy; 4 Dipartimento di Fisica, Università di Pisa, Largo B. Pontecorvo 3, 56127 Pisa, Italy; alessandro.pitanti@unipi.it (A.P.); leonardo.vicarelli@unipi.it (L.V.); 5 National Enterprises for nanoScience and nanoTechnology (NEST), CNR—Istituto Nanoscienze, Piazza San Silvestro 12, 56127 Pisa, Italy; 6 Dipartimento di Ingegneria Industriale, Elettronica e Meccanica, Università degli Studi Roma Tre, Via della Vasca Navale 84, 00146 Roma, Italy; monica.lamura@uniroma3.it; 7 Dipartimento di Ingegneria dell’Informazione ed Elettrica e Matematica Applicata, Università degli Studi di Salerno, 84084 Fisciano, Italy; 8 Microsystems Technology (MST), Fondazione Bruno Kessler (FBK), Via S. Croce 77, 38122 Trento, Italy; bagolini@fbk.eu; 9 Istituto Nazionale di Fisica Nucleare (INFN) Sezione di Trieste, Via Valerio 2, 34127 Trieste, Italy

**Keywords:** microelectromechanical systems (MEMSs), field-programmable gate array (FPGA), lock-in amplifier, terahertz detection and imaging

## Abstract

Field programmable gate arrays (FPGAs) have not only enhanced traditional sensing methods, such as pixel detection (CCD and CMOS), but also enabled the development of innovative approaches with significant potential for particle detection. This is particularly relevant in terahertz (THz) ray detection, where microbolometer-based focal plane arrays (FPAs) using microelectromechanical (MEMS) resonators are among the most promising solutions. Designing high-performance, high-pixel-density sensors is challenging without FPGAs, which are crucial for deterministic parallel processing, fast ADC/DAC control, and handling large data throughput. This paper presents a MEMS-resonator detector, fully managed via an FPGA, capable of controlling pixel excitation and tracking resonance-frequency shifts due to radiation using parallel digital lock-in amplifiers. The innovative FPGA architecture, based on a lock-in matrix, enhances the open-loop readout technique by a factor of 32. Measurements were performed on a frequency-multiplexed, 256-pixel sensor designed for imaging applications.

## 1. Introduction

Terahertz (THz) radiation is utilized across multiple sectors, encompassing both basic research and commercially impactful applications. For example, such radiation is employed in the telecommunications industry [1,2] and in astronomy, where it provides crucial insights into astronomical phenomena such as galaxy formation [3] or the detection of carbon for the investigation of extraterrestrial life forms. Additionally, in medical diagnostics, terahertz waves enable non-invasive imaging techniques capable of detecting early-stage skin cancer or analyzing the composition of pharmaceutical drugs [4,5]. Driven by these potential applications, THz imaging science and technology, spanning the 0.1-to-10 THz range, has made impressive progress in recent years [6]. However, despite these numerous applications, the widespread adoption of THz technology still faces challenges, with many detection approaches remaining in the research phase.

Recent works have explored the use of micro-electro-mechanical systems (MEMSs) to address challenges in terahertz radiation detection [7]. MEMS technology is highly versatile for developing innovative sensors across various fields, offering advantages such as low power consumption, simple integration, and overall flexibility. However, one of the main hurdles in realizing a MEMS-based THz imaging detector is the fast addressing of a large number of devices, which requires both responsive sensors and scalable readout techniques. Research into entirely new architectures is highly active, with recent solutions investigating approaches such as optical reflectivity/diffraction and electrical resistive readouts [8].

Another commonly adopted technique for addressing multiple devices is multiplexing. Among the various types of multiplexing, frequency-multiplexed schemes are particularly effective, allowing interaction with individual devices by subdividing the communication medium’s bandwidth into smaller, non-overlapping frequency bands. For instance, in an array of MEMS resonators with distinct resonant frequencies, each band corresponds to a range of frequencies centered around each resonator’s original resonance [9,10]. Finally, in order to keep the dimensions of the system compact despite the increase in the number of devices, fully electronic actuation and sensing are required [11].

Following these criteria, previous works [12,13] have discussed the possibility of exploiting this technique for the readout of electrically addressable micromechanical trampoline resonators (MTRs). Each MTR is characterized by a uniquely engineered resonance frequency, which changes slightly with the absorption of radiation across a wide spectral range (from visible to sub-THz). The resonance frequency shift is determined by the thermally induced compressive stress due to the resonator thermal expansion inside the fixed frame [14]. Both actuation and sensing are obtained thanks to two golden electrodes deposited on the surface of the devices. Building upon these studies, a new architecture has recently been proposed [15], which is highly scalable and capable of simultaneously handling a much larger number of resonators.

As will be discussed later in this work, a phase-sensitive detector is required to measure the response of each individual device within a frequency-multiplexed scheme. This type of instrument, known as a lock-in amplifier, operates by comparing a reference signal with an incoming signal, allowing it to isolate single tones in the response with a complex spectrum. Commonly available lock-in amplifiers are not capable of performing advanced processing, and due to the limited number of channels, a setup based solely on these instruments would be highly impractical. Hence, alternative readout electronics and fast processing are key to fully obtaining compact and flexible solutions [16,17]. Specifically, the previously described frequency-multiplexed readout technique can be paired with programmable logic devices, such as FPGAs, which can process all the information from individual sensors in parallel, resulting in an innovative readout that could potentially lead to a new fast and versatile imaging device.

Thanks to the distinct resonances of the MTRs, it is possible to generate a signal with multiple frequency components, enabling the simultaneous actuation of several devices across a single communication channel. By leveraging the FPGA’s parallel computing capabilities, a multi-frequency capable lock-in readout is implemented [18]. This unique multi-frequency addressing is ideally suited for FPGAs, which can efficiently handle and process data from multiple sensors in parallel while simultaneously communicating with a host PC.

In this paper, we present an FPGA architecture capable of independently sweeping eight tones. The integration of 32 custom digital lock-in amplifiers within an Intel Cyclone^®^ V (Intel, Santa Clara, CA, USA) enables the parallel readout of an equal number of MTRs by transmitting polytonal signals across four lines simultaneously.

We begin with a general overview of the sensor array that constitutes the entire detector, covering both the electromechanical and electronic components. Next, we delve into the operations required to control the sensors and acquire their signals, detailing how these tasks are executed within the FPGA. Finally, we present the results obtained by illuminating a 16 × 16 array of MTRs with both a 445 nm laser and a 100 GHz source, highlighting the most critical aspects of the electronic section’s performance in each case.

## 2. Materials and Method

Thanks to frequency multiplexing, which enables interaction with multiple devices through a single common communication medium, the presented design is both compact and easily scalable. To better understand how this multiplexing is utilized, the following sections will provide a brief description of the MTRs, focusing on the operating principles and radiation detection. Subsequently, we will describe the experimental setup, focusing on the readout scheme and front-end electronics. Finally, we will present a comprehensive overview of the FPGA design, with particular emphasis on the custom lock-in detection core and its application for polytonal excitation.

### 2.1. Micromechanical Trampoline Resonator

**Figure 1:**
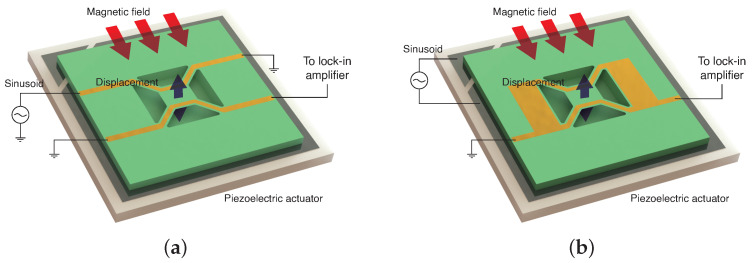
(**a**) Readout scheme of an MTR with two golden tracks where the displacement is induced via Lorentz’s force due to current injection. (**b**) Readout scheme of an MTR with two golden tracks connected in parallel. In this case, the displacement is induced via a piezoelectric actuator underneath the device.

A single MTR consists of a microfabricated silicon nitride (Si_3_N_4_) suspended membrane anchored to the substrate via four tethers. A detailed characterization of an individual device can be found in previous works [12,19], where membrane vibrations are induced using a piezoelectric actuator, and the response is measured through a self-mixing interferometric apparatus. Improving upon this approach, full electrical control of the devices has been recently achieved and studied [15]. Two gold electrodes run through the tethers of the resonators, and the chip containing the devices is placed within a static magnetic field. This magnetic field transduces the membrane’s movement into an electrical current in the gold tracks; conversely, injecting a current through a track generates force that can be used to actuate the membranes, as in Figure 1a. Thanks to this approach the precise control of the coherent vibration of single MTRs is achieved [15].

Another viable approach, depicted in Figure 1b, is to use a piezoelectric actuator to move the entire chip at once while measuring the current from both electrodes. This configuration enables the measurement of signals from an entire column of the array, even if one of the two branches is interrupted. Furthermore, although this method was not employed in the experiments discussed in this paper, with appropriate termination of the branches, the piezoelectric vibration enables a differential reading of the signals, potentially improving signal quality and noise rejection.

The series connection of multiple devices, first introduced in [15], enables the use of frequency multiplexing, allowing multiple devices to be read with just two electrodes. During the design process, the geometry and other characteristics of the chip—such as tensile stress and tether dimensions—are carefully adjusted to prevent potential resonance overlap among devices sharing the same connection.

Control of the devices through this connection can be applied both in an open-loop and in a closed-loop configuration [20]. In an open-loop configuration, a chirp signal—a sinusoidal signal with a linearly increasing frequency—is generated, spanning the entire range of interest. The lock-in amplifier detects higher amplitudes each time the frequency sweep reaches the resonance of an MTR. The frequency shifts of these measured peaks, relative to the resonances observed under dark conditions, are proportional to the radiation incident on each membrane [12]. This detection method enables the scanning of a large number of devices at a nearly constant rate while maintaining simpler control logic and relying on subsequent signal-processing algorithms to identify the peaks and measure the resonances.

In contrast, closed-loop control leverages phase-locked loop (PLL) detection. By utilizing the phase information calculated using the lock-in amplifier, a phase-sensitive circuit measures the phase difference relative to the resonance peak, enabling the control system to adjust the generated frequency accordingly and thereby tracking each device’s resonance. As the control system directly calculates frequency shifts, this approach allows for the precise measurement of extremely rapid changes in the devices’ responses.

Although the automatic tracking of the devices’ resonance is theoretically faster, it requires additional logic and increases complexity, which, in turn, reduces the number of phase-sensitive detectors that can be synthesized in the FPGA with a given amount of resources. Furthermore, the complex phase response generated via multiple devices and their non-linearities [15] can occasionally cause PLL circuits to lose track of resonance. In such cases, an algorithm is needed to sweep the frequency and find the correct phase to “lock” back onto the resonance, further complicating the control logic.

### 2.2. Readout Platform

**Figure 2:**
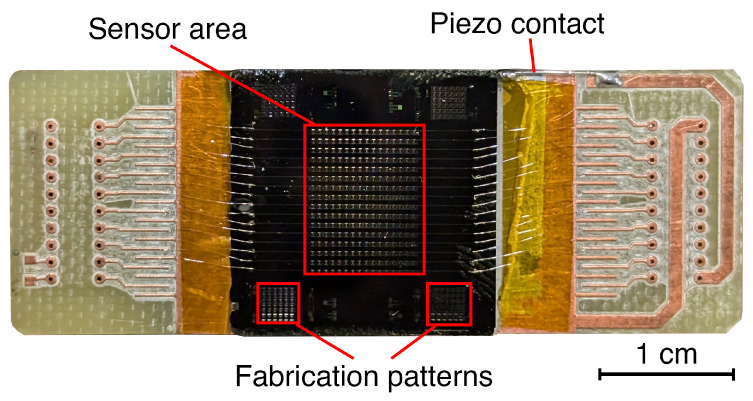
Image of the 16 × 16 sensor glued to a piezoelectric actuator.

The sensor used in this work is a bidimensional array of 16 × 16 MTRs (∼1.1 × 0.8 cm^2^). Although connecting all 256 in a series is feasible, the frequency shifts generated due to radiation absorption, which can measure up to a couple of kHz, could lead to the potential overlapping of multiple MTRs’ responses. For this reason, an alternative configuration is proposed. The array, depicted in Figure 2, is subdivided into 16 columns, each connected with its own couple of wires. The resonances of the MTRs are similar for each column, allowing for the actuation of multiple devices in parallel but requiring separate readout circuits.

The diversification of the resonant frequency across the pixels of a column is achieved by adjusting the tether width (*W*) of each device. Increasing *W* raises the trampoline’s flexural rigidity, resulting in a higher fundamental resonant frequency ($f_{0}$). This allows for a fine-tuning of the resonances for each pixel, ensuring no overlap between the highest and lowest frequencies, with the maximum tether width not exceeding the second excited mode of the smallest *W*.

A semi-empirical analytical relation for $f_{0}$ and *W* is derived from finite-element analysis of a 100 μm-wide, 300 nm-thick Si_3_N_4_ membrane under 0.29 GPa residual stress, suspended over a 300 μm cavity [21]. The analysis includes a prestressed eigenfrequency analysis with thermo-mechanical coupling at 293 K. These simulations highlight a monotonic, positive, nonlinear dependence between $f_{0}$ and *W*, which can be accurately modeled by a power-law relation:

(1) $$f_{0}=aW^{b}+c$$

**Figure 3:**
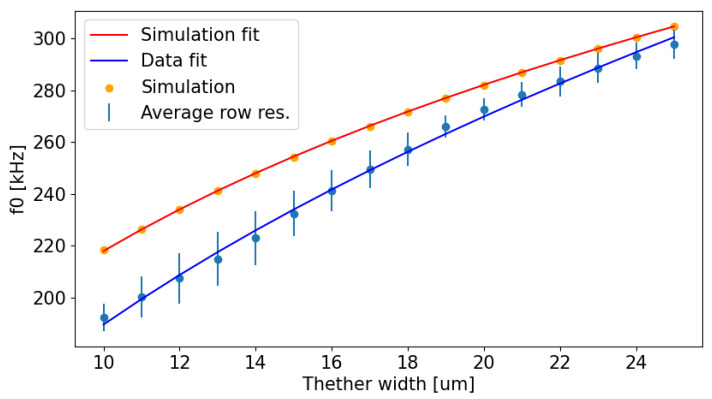
Comparison between the simulated and measured $f_{0}$ as a function of *W* and the respective power-law fits.

During the manufacturing of the devices, fabrication constraints require the setting of the minimum tether width at 10 μm. The resonant frequencies of the devices are varied by increasing *W* in 1 μm increments up to 25 μm. Figure 3 illustrates a comparison between the simulated fundamental resonant frequency, $f_{0}$, and the average resonant frequency measured along the rows of the chip as a function of *W*.

Discrepancies between the simulated and measured resonant frequencies are observed, especially for lower values of *W*. Specifically, the simulated frequencies range from 218.4 kHz (*W* = 10 μm) to 304.8 kHz (*W* = 25 μm), while the actual measured frequencies span from approximately 192.3 kHz to 297.8 kHz. These differences can likely be attributed to non-idealities in the fabrication process, such as variations in material properties, tether dimensions, and residual stress. Additionally, the finite element simulations do not account for damping effects, which can also contribute to the observed discrepancies between a simulation and experimental results. Despite the slight mismatch of the results, the simulations still turn out to be helpful in the design of the sensor and the mechanical characterization of the MTRs.

**Figure 4:**
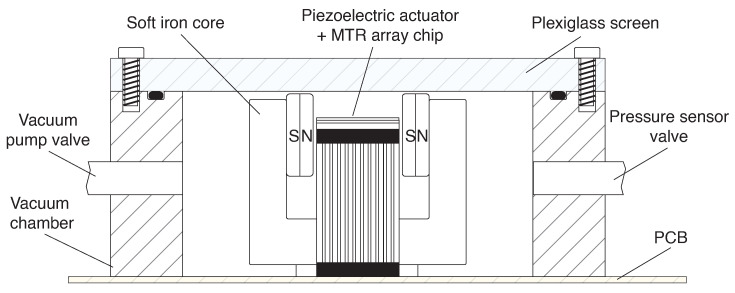
Schematic representation of the vacuum chamber hosting the devices.

As previously mentioned, the vibrational motion of the MTRs is converted into a current, thanks to a 0.25 T magnetic field generated via a pair of Nd magnets positioned on a C-shaped soft iron core. Moreover, in order to increase the quality factor of the devices, allowing for the easy measurement of the resonances, the entire chip is mounted inside a vacuum chamber. In moderate vacuum conditions (10^−2^ mbar), this produces Q-factors > 10,000. A schematic representation of the chamber is depicted in Figure 4.

To actuate and read these devices, a partially custom FPGA-based electronic system was devised and employed. This system was designed with a modular approach for maximum flexibility, allowing the easy improvement and upgrading of single sections following the evolution of the bolometers. This approach is necessary due to the novelty and rapid development of these devices, for which different readout and actuation techniques must be developed and tested: a task for which the computational power and high reconfigurability of FPGAs are necessary.

The current version of the electronic hardware used to study these innovative sensors is composed of 3 printed circuit boards (PCBs). The main PCB is a “Cyclone^®^ V GX Starter Kit” evaluation board (EVB) from Terasic, housing an Altera Cyclone^®^ V FPGA (5CGXFC5C6F27C7) and various peripherals, which are mostly unused in our system, apart from the switches, buttons, and LEDs used as a status control and an indicator. The EVB connects through a high-speed mezzanine card (HSMC) connector to a custom-made PCB equipped with the following:

- One small form-factor pluggable (SFP) connector for a gigabit communication with a host personal computer (PC);
- Four 16-bit digital-to-analog converters (DACs) with a 5V single-ended output operating at ∼1.43 MS/s (AD5541);
- One 4-channel, 16-bit analog-to-digital converter (ADC) with a ±1 V input operating at 50 MS/s (AD9553);
- Thirty-four digital input/outputs (IOs) with 2.5 V or 3.3 V levels.

Finally, a custom conditioning PCB was designed to interface the HSMC PCB with the devices. This PCB performs three main functions:

- Removing the offset from the DAC signal in order to obtain 3 independent buffered outputs with a ±2.5 V range;
- Multiplexing the 16 inputs to 4 signals for the ADCs and amplifying them by a factor of 10, using 2 dual-channel analog multiplexers (DG4052E) controlled via the HSMC digital IOs;
- Providing some digital IOs in order to trigger external sources to obtain modulated experiments.

**Figure 5:**
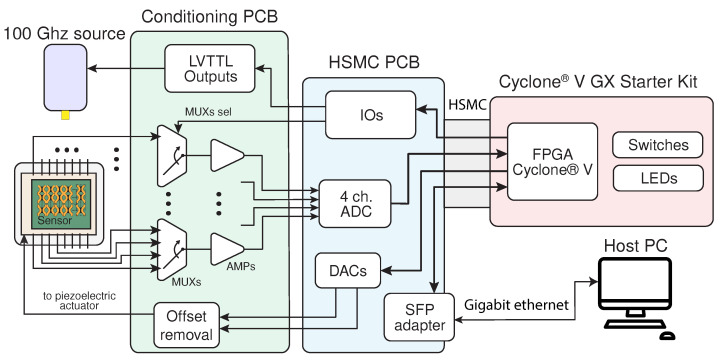
Block diagram of the developed readout platform, composed of 3 PCBs, the bolometer matrix, a radiation source, and the host PC controlling the acquisition.

The resulting electronic system is capable of providing 3 independent actuation signals to the bolometers, and it can be connected to up to 16 bolometer arrays, acquiring 4 of them at the same time. A block diagram of the hardware composing this readout platform and its interconnection for a typical imaging experiment is reported in Figure 5.

Leveraging the designed mechanical and electrical hardware, an open-loop readout scheme was developed. While a close-loop readout is capable of real-time tracking and usually achieves higher accuracy, it is currently not suitable for our emerging technological platform. In fact, in the present configuration, our platform uses single actuation for a whole 2D array, and a PLL locked on a membrane would interfere with membranes on other rows but with a similar frequency, not allowing for row-wise parallelization. Moreover, closed-loop techniques for multiple MEMSs require a PLL for each device [22], which is difficult to scale, or a periodic PLL unlocking and locking [23], which is slow in high-Q-factor resonators, thus proving difficult for our 16 × 16 matrix. On the other hand, an open-loop readout scheme can, with a single sweep, actuate all the membranes whose frequency response can be read on 4 columns at the time using the 4 ADC channels, allowing for the measurement of more devices in parallel at a constant rate.

### 2.3. FPGA Design

**Figure 6:**
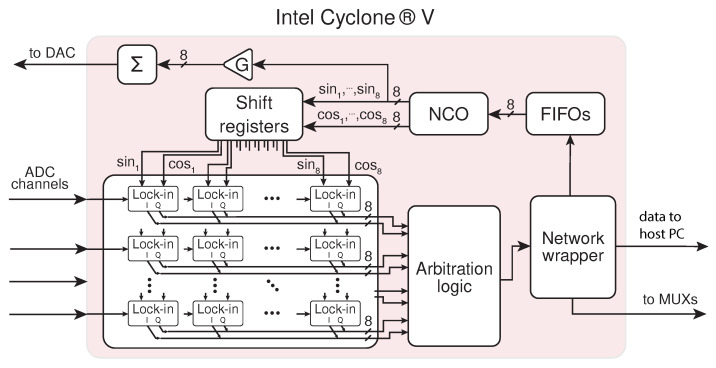
Block diagram of the implemented FPGA firmware highlighting the main modules and their interconnections.

The FPGA firmware’s main tasks are to actuate the resonators, through a DAC driving the piezoelectric actuator, and to measure the signals coming from the resonators through the four channels of the ADC. A block diagram of the FPGA firmware logic is reported in Figure 6.

As previously suggested, the signal processing performed by the FPGA is a lock-in-based, open-loop approach: at first, a commercial lock-in amplifier was used to measure one column at a time, but it was found to be unsuitable for evaluating multiple frequency shifts within a bidimensional configuration. Thanks to the 4-channel ADC used and the FPGA parallelization capability, the open-loop readout of the whole 2D matrix can be sped up by employing 4 parallel lock-ins, all implemented inside the FPGA. In this case, all the lock-ins use the same reference frequency, which is also sent to the piezoelectric actuator, but on different input signals. This allows for a reduction by a factor 4 of the time required for a complete matrix readout.

To further reduce the acquisition time, the actuation was also parallelized: by using *N* numerically controlled oscillators (NCOs) and adding their outputs together, it is possible to generate multi-frequency actuation, which we will refer to as “polytonal”. This allows for the performance of a frequency sweep *N* times faster by separating it in *N* smaller sweeps executed at the same time. To properly demodulate the resonators signal, the number of lock-ins must also be increased by a factor, *N*, one for each reference frequency generated via the NCOs. In order to use the polytonal approach, the *N* generated frequencies must be separated enough to allow the low-pass filter (LPF) of each lock-in to remove the contributions of the other NCOs. Moreover, the sweep frequencies must be such that even the harmonics do not interfere with the fundamentals: this is not a concern in our application since the resonators are designed to avoid harmonics’ overlap. The developed FPGA firmware takes advantage of this polytonal technique using 8 NCOs. This, together with the multi-channel ADC, requires the implementation of 32 independent lock-in amplifiers but allows for a reduction in the acquisition time of the whole 2D matrix by a factor of 32.

The firmware is controlled via the host PC through a custom hardware description language (HDL) module implementing multi-port UDP communication. This module parses the frequency sweeps’ parameters (e.g., start frequency, frequency step, and duration) and stores them in 8 independent FIFOs for the NCOs. This FIFO-based approach allows for the scheduling of multiple sequential sweeps, and it is useful to obtain a piecewise frequency response around frequencies of interest, for example by generating frequency sweeps only around the membranes resonances and not the whole spectrum, thus reducing the acquisition time.

While the lock-in implementation and the NCO/actuation logic will be discussed in greater detail in Section 2.3.1 and Section 2.3.2, respectively, it is important to note that the NCOs provide the reference signal for the lock-ins through a RAM-based shift register. The use of this shift register is necessary to compensate for the time delay introduced via the DAC, ADC, and related firmware between the NCO and the lock-in input. This time delay, if not accounted for in the reference signal, will otherwise introduce a linear phase shift with a frequency in the measured bolometers’ frequency response.

The lock-ins outputs are sampled once per frequency step, after the lock-in filter settling time, and they are stored in a single FIFO using round-robin arbitration logic. The sampled I/Q values are transmitted to the host PC for post-processing and visualization.

#### 2.3.1. Lock-In Design

A lock-in amplifier is essentially a homodyne receiver used to extract the signal amplitude and phase in a noisy environment. To achieve this, a lock-in requires a reference signal, as well as its 90° shifted version, to measure the in-phase and quadrature components of the input signal [24].

**Figure 7:**
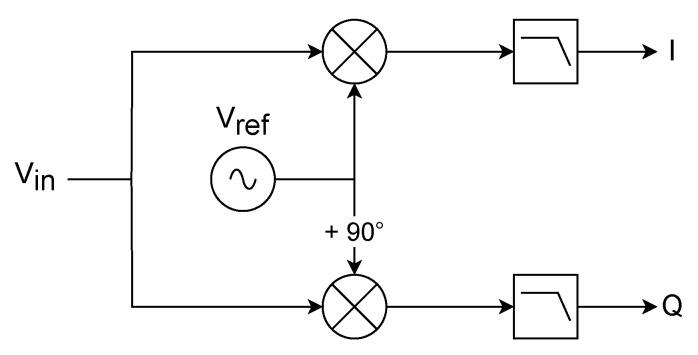
Representation of a lock-in amplifier structure composed of 2 parallel paths made using a mixer and a low-pass filter.

The structure of a lock-in amplifier, depicted in Figure 7, is composed of 2 parallel processing paths. The first element of each path is a mixer, used to down-convert the input signal with a reference signal, or its 90° shifted version. The mixer produces both a baseband component and an unwanted one at twice the frequency of the reference signal. The low-pass filter (LPF), typically with a very narrow bandwidth, is necessary to remove the unwanted signal generated via the mixer and to reduce the output noise. The 90° shift in the reference signal between the two paths allows for the obtaining of the in-phase (I) and in-quadrature (Q) components. Usually, even when implemented digitally, the output LPFs are equivalent to one or more stages of single-pole ”RC” filters [24].

While it is straightforward to see that a mixer requires a multiplier, the filter implementation can change drastically with a trade-off between performance and resource utilization, which is a limiting factor in FPGA firmware design. Although finite impulse response (FIR) filters are straightforward to design with an arbitrary frequency response and guaranteed stability, they typically require a high number of coefficients to achieve a sharp cut-off, making them unsuitable for our resource-limited application, where many lock-ins are needed. On the other hand, infinite impulse response (IIR) filters require a careful design to avoid instability, but they require many fewer coefficients to achieve a sharp cut-off than equivalent FIR filters.

It is important to note that, while there are some techniques to reduce the number of multipliers of coefficients for an FIR filter, such as multistage filtering [25], polyphase filtering [26], MAC FIR filters [27], and sparse FIR filters [28], these solutions typically limit the flexibility of the filter to some specific coefficient sets or the achievable filter bandwidth. This is also true for multiplierless FIR and IIR filters [29], which are limited to subsets of coefficients (typically powers of 1/2) or require dedicated HDL implementation for a given bandwidth. Since our sensors are constantly evolving and under investigation, strong flexibility in the filter bandwidth is needed, making all of these techniques unsuitable.

In order to properly emulate a lock-in filter while also reducing the number of multipliers needed, a single-pole IIR filter emulating a classic “RC”-style filter was designed, and it can be cascaded to achieve higher-order filtering. When starting from an analog RC filter, the equivalent digital transfer function can easily be obtained through a bilinear z-transform [30]:

(2) $$H\left(s\right)=\frac{1}{1+s\tau }⟹H\left(z\right)=\frac{\alpha }{2}\frac{1+z^{-1}}{1-\left(1-\alpha \right)z^{-1}}\;\mathrm{where}\;\alpha =\frac{2}{1+\tau }.$$

**Figure 8:**
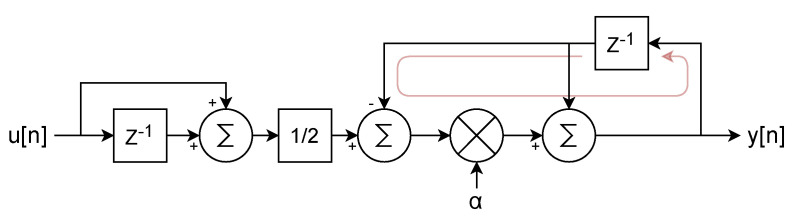
Diagram of the single-pole IIR LPF filter described by the difference Equation (4), with the critical path highlighted in light red.

The derived H(z) is equivalent to a canonical first-order, low-pass IIR digital filter [31], whose analog −3 dB cut-off frequency, $f_{c}$, can be set by means of α as follows:

(3) $$\alpha =1-\frac{1-\sin\left(\omega_{c}\right)}{\cos\left(\omega_{c}\right)},\;\mathrm{where}\;\omega_{c}=2\pi \frac{f_{c}}{f_{s}}$$

and $f_{s}$ is the sampling frequency, which is 50 MHz in our system. The transfer function in Equation (2) provides the difference equation

(4) $$y\left[n\right]=\left(1-\alpha \right)y\left[n-1\right]+\frac{\alpha }{2}\left(u\left[n\right]+u\left[n-1\right]\right),$$

that can be implemented in FPGA, following the diagram represented in Figure 8.

While the filter proposed in Figure 8 is easy to implement, it presents two drawbacks for our specific application. First, using a multiplier for each filter stage would require 128 DSP blocks to support 32 lock-ins. Second, the long critical path [32]—comprising two adders and a multiplier—limits the achievable clock rate of the filter.

To improve the filter design and reduce resources, we used a C-slow implementation, also called multichannel interleaving [32]. This technique replaces each register with *C* registers, which can be retimed to improve the critical path and operate on *C* data streams that need to be multiplexed and demultiplexed.

**Figure 9:**
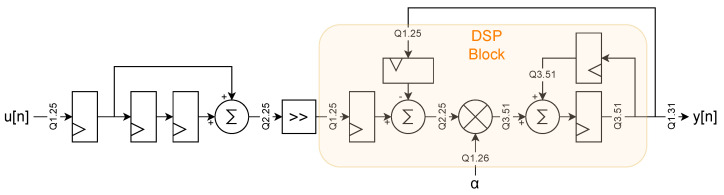
Diagram of the 2-slow implementation of the single-pole IIR LPF filter, with the DSP block hardware highlighted in orange.

The resulting two-slow filter, implemented in FPGA, is reported in Figure 9. Here, the diagram reports the fixed-point format used with the Q notation and the logic embedded in the Cyclone^®^ V Variable Precision DSP Block [33]. The bit width for the multiplier inputs allows for the maximization of the usage of a single DSP block, capable of one 27 × 27 multiplication or two 18 × 18 ones, and using a Q1.31 fixed-point value for the output guarantees a sub-nV resolution for the I and Q component (with a ±1 V input). Moreover, while the implementation in Figure 9 still presents a critical path made by 2 adders and a multiplier, the represented implementation takes full advantage of the hardware in the DSP block, using the input registers, pre-adder, multiplier, accumulator, output register, and double accumulation register [33] and thus allowing for the achievement of a reported maximum operating frequency ($f_{max}$) greater than 250 MHz. While this $f_{max}$ is far above the 100 MHz needed for a 50 MS/s data rate, other implementations (i.e., with the accumulator feedback register outside the DSP block) struggled to meet the requirements. Even the hardware realization of Figure 8 struggled to reach the needed 50 MHz clock due to poor DSP-register usage. This underlines that hardware-aware design is important to developing efficient FPGA firmware.

In our case, the use of a 2-slow implementation allowed us to use the same filter and mixer for both I and Q components, alternating them.

**Figure 10:**
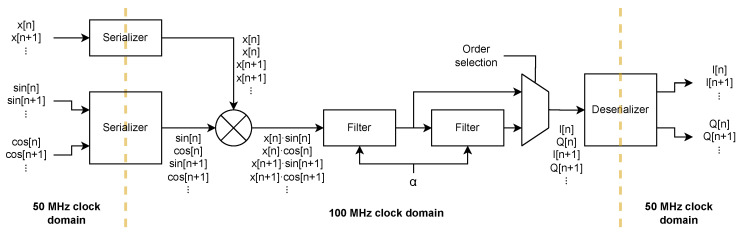
Block diagram of the 2-slow implementation of the lock-in with selectable order.

The 2-slow approach was extended to the mixer as well, allowing a single multiplier to be used for both the sine and cosine multiplications. The lock-in block diagram of the proposed implementation is reported in Figure 10. As previously suggested, the 2-slow implementation requires the clock rate to be twice the data rate, and since the ADC data rate is 50 MS/s, a 100 MHz clock, synchronous to the 50 MHz one, is generated through a phase-locked loop (PLL) to drive the filter and the mixer.

#### 2.3.2. Polytonal Excitation

The actuation signal for the membranes is generated via the NCO IP Core from Intel, which is configured for a dual-channel output, to obtain both the sine and cosine, and for multichannel operation. In multichannel operation, similarly to the multichannel interleaving described for the lock-in filter, the IP core input and output are sequentially multiplexed and demultiplexed to obtain 8 parallel sin/cos pairs of arbitrary frequency and unitary amplitude. While these pairs are used in the 32 lock-ins as the references for the IQ demodulation, the 8 sines are also used for the actuation. The sines are independently scaled to achieve arbitrary spectral density and are summed together. The arbitrary scaling of the NCO outputs is useful to compensate for a non-flat transfer function in piezoelectric actuators.

This polytonal signal is low-pass filtered through a 6th order IIR filter, composed by 3 of the same 2-slow filters previously described, with a 500 kHz bandwidth. This filter acts as an anti-aliasing filter since the polytonal signal needs to be downsampled to ∼1.43 MS/s to accomodate the DACs’ data rate.

**Figure 11:**
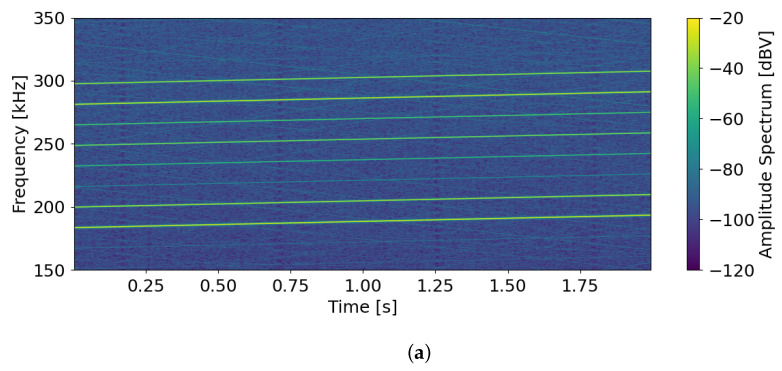

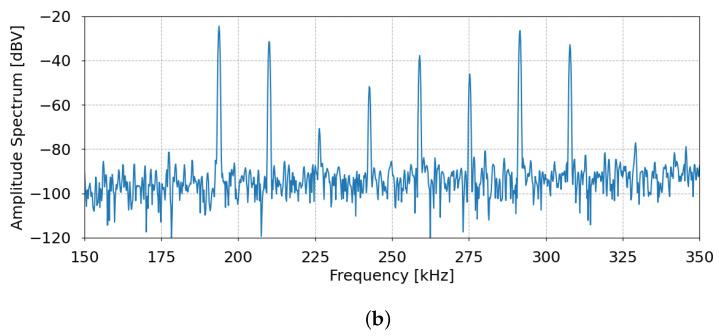
(**a**) Spectrogram of a polytonal excitation signal with the tones being equally spaced in frequency and with arbitrary amplitudes between 100 mV and 0.5 mV. (**b**) Amplitude spectrum of the signal at 2 s.

In Figure 11a, a portion of a spectrogram of a polytonal excitation signal generated via the NCOs is reported, with the tones being equally spaced in frequency and with arbitrary amplitudes between 100 mV and 0.5 mV. Figure 11b shows the amplitude spectrum of the signal in Figure 11a at time 2 s in order to better visualize the different amplitudes.

## 3. Experimental Setup and Results

**Figure 12:**
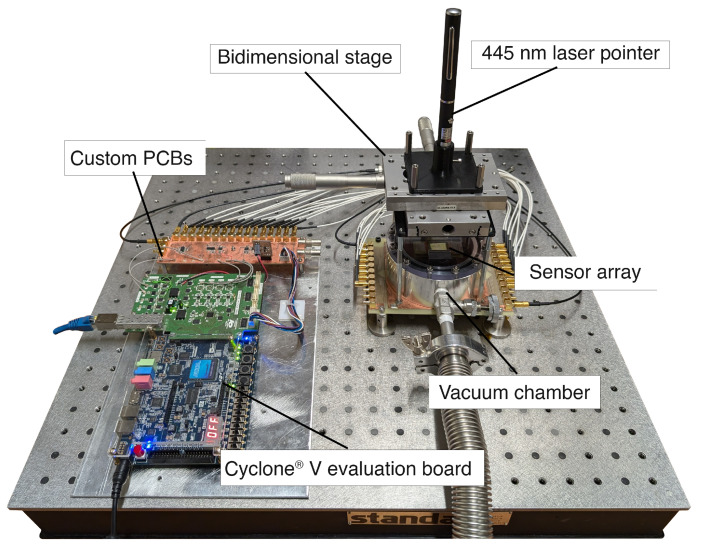
Picture of the experimental setup for laser pointer-based measurement, including the vacuum chamber housing the sensor and the readout electronics.

To assess the efficacy of the readout platform and the MTRs’ responsiveness, measurements were conducted using a 445 nm laser pointer and sub-terahertz radiation sources. The laser pointer enables focused acquisition on individual membranes, allowing for the calibration of individual responses and verification of the frequency-multiplexed acquisition with polytonal excitation. In contrast, a sub-terahertz source is employed to capture actual images with the sensor, providing insights into macroscopic effects. In both measurement setups, a bi-directional linear translation stage with micrometric precision is used to move either the source or a mask. A picture of the experimental setup, for a laser pointer-based measurement, is reported in Figure 12.

The amplitude and phase measured via each lock-in amplifier, along with the reference frequency, are transmitted to the host PC via a gigabit Ethernet connection. On the PC, LabVIEW-based software (LabVIEW Developement System 18 Q1, National Instruments, Austin, TX, USA) is employed to coordinate the acquisition process and save the data associated with the frequency sweeps. For these initial results utilizing the frequency-multiplexed readout approach, the resonances are identified by calculating the local maxima of the absolute value of the amplitude’s derivative.

### 3.1. Laser Pointer (445 nm)

**Figure 13:**
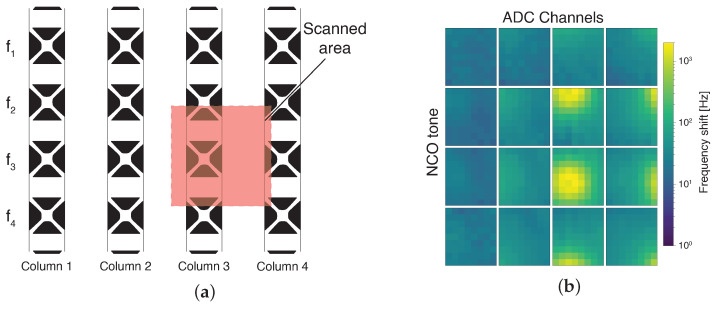
(**a**) Schematic representation of the area scanned relative to the measured MTRs’ position. (**b**) Frequency shift relative to the dark acquisition of the 16 MTRs. Log scale is used to better highlight the response of non-directly illuminated MTRs.

The laser pointer used for the single-membrane characterization is a laser pointer with a wavelength of 445 nm. A pinhole is used to reduce the spot size on the sample surface to a ∼500 μm diameter, with an incident power of ∼600 uW. As mentioned, the aim of this setup is to characterize the MTRs in terms of their frequency shifts. For this first set of measurements, the laser is mounted on the linear translation stage and is moved with 100 μm steps to cover an area of 0.9 × 0.9 mm^2^, resulting in a 9 × 9 matrix. Since the inter-axial distance between two membranes measures 0.5 and 0.7 mm along the rows and columns, respectively, as shown in Figure 13a, the aforementioned scan is guaranteed to directly hit an entire membrane. For each position, a 4-tone frequency sweep is generated, allowing for the measurement of a total of 16 devices in parallel, thanks to the four ADC channels. This allows for an evaluation of the proper functioning of the polytonal sweep and an evaluation of the MTRs’ responses at different distances from the spot. For this measurement, the sweep step is set to 2 Hz, with 20 ms per step in order to reduce the noise. Using these parameters, each generated tone is capable of sweeping a 2 kHz span in 20 s.

Figure 13b illustrates the frequency shifts (relative to the dark acquisition) observed in the 16 MTRs as a function of the laser pointer’s position. The devices directly exposed to the laser spot, clearly visible in the bottom right, exhibit significant frequency shifts, with values of several hundreds of Hertz and a maximum shift of 2 kHz. Due to the difference in the measurements of these devices, a logarithmic color map is used to better appreciate the contribution of each MTR.

These measurements indicate that the frequency multiplexing readout scheme and the sensing of multiple devices on different connections—even those with similar resonance frequencies—are indeed viable techniques. This validation supports the potential for developing more efficient readout architectures using these methods. The ability to handle multiple devices simultaneously while maintaining accuracy in their responses confirms the effectiveness of the proposed approach for scalable and high-speed sensor arrays.

### 3.2. Sub-THz Source (0.1 THz)

**Figure 14:**
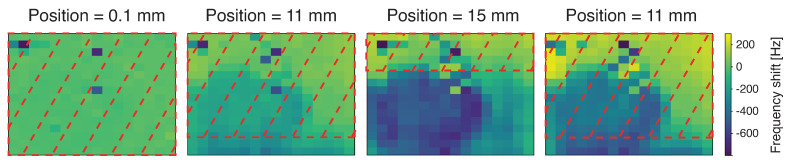
Image obtained with the MTRs sensor using a 0.1 THz source and a copper blade in different positions. The pixels inside the red dashed rectangle are covered by the mask. The last frame shows that the unwanted feature remains upon coverage.

For the measurements with the 0.1-THz source (manufactured by Terasense), a thin copper edge is mounted on the translation stage and is used as a mask. The source has a power of about 30 mW and is placed 4 cm above the sensor, ensuring that all the MTRs are illuminated. To obtain the dark measurement, the mask is positioned such that all the devices are covered. Then, thanks to the translation stage, the mask is moved with 1 mm steps, exposing a wider area of the sensor. This process is repeated until half of the sensor is illuminated, and then the blade is moved back, still with 1 mm steps. Figure 14 depicts the frequency shifts of the array with respect to the dark measurement with the mask in four different positions.

To enhance the acquisition speed, all 32 lock-in amplifiers were used, along with optimized sweep parameters: a frequency step of 5 Hz and a timestep of 10 ms. Given that the MTRs’ resonances span a range of 120 kHz, this range was divided among the 8 tones of the polytonal sweep, resulting in 8 equal sub-intervals of 15 kHz each. With these settings, a complete sweep takes 30 s, translating to approximately 0.5 s per device (since each line contains 16 MTRs, and the setup scans 4 lines simultaneously). Consequently, a full image can be acquired in 2 min, with the time taken for the multiplexer switch and UDP communication being negligible.

Clearly, when compared with other imaging devices, the time intervals required for this sensor are relatively long. However, as previously discussed, the design is highly scalable, and by integrating additional lock-in amplifiers, it is possible to achieve even faster frame rates. This scalability opens the door to significantly improved acquisition speeds in future iterations of the device, making them more competitive with existing imaging technologies.

Regarding the measurement, a circular spot was immediately noticeable in the bottom left corner of the image. Upon further inspection, it was determined that this feature is not caused by direct illumination from the source. In fact, the spot persists in the same position when the sensor is illuminated with the laser pointer. This suggests that the spot is most likely due to uneven thermal dissipation between the chip and the piezoelectric actuator, rather than the source itself. Additionally, discontinuities are visible in the central columns of the detector. The MTRs in these rows exhibited a very low signal-to-noise ratio, and in some cases, not all resonances were distinguishable. This can be attributed to the lower magnetic field in the middle of the sensor. Furthermore, other discontinuities, observed in particular in the uppermost part of the sensor, suggest possible imperfections in the fabrication, which lead to overlapping frequencies. This needs to be addressed for more consistent results across the entire sensor array.

Despite the imperfections observed in the central columns and the presence of the unwanted circular spot, the rightmost lines of the detector, which are not directly affected by the spot, show clear resonance shifts. In these areas, it is possible to distinguish the portion of the sensor that is not covered by the mask. The resonance shifts in this unmasked region are significant, measuring in the hundreds of Hertz, with a maximum shift of 300 Hz. This indicates that, even with the observed issues, the system is still capable of detecting and differentiating between covered and uncovered regions of the sensor, demonstrating its potential for effective sub-THz imaging applications.

## 4. Discussions and State-of-the-Art Comparison

The observed shifts in the measurements differ significantly between the two readiation sources. In particular, the shift produced using the 445 nm laser is considerably larger. This can be attributed to the different surface power densities hitting the single membranes. However, one important aspect to consider is that the membranes exhibit varying absorption characteristics at different wavelengths. In fact, recent studies have demonstrated that absorption in the near-IR and THz bands can be significantly enhanced by depositing specific materials on the membranes’ surfaces [34].

In a classic open-loop configuration, the time required to measure each device depends on the frequency sweep steps, which determine the sensor’s resolution, and the time spent on each step, with longer times leading to better noise rejection. Beyond optimizing these parameters according to the setup conditions, further enhancements can be achieved through advanced sweep logic, such as concentrating the frequency sweeps in regions containing all the peaks of a single row while accounting for the maximum frequency shift of the devices. However, the potential improvements achieved this way are rather limited.

Conversely, by leveraging the superposition of eight independent tones generated via the NCO and an ADC with four channels, 32 parallel data streams are generated, significantly boosting the acquisition speed. Additionally, the presented approach is easily scalable, allowing for a further enhancement of the system’s capabilities. As shown in Table 1, since each lock-in circuit requires 3 DSP blocks, the primary bottleneck of the design described in this paper is the number of DSP blocks available in the Cyclone^®^ FPGA and the number of tones that can be generated via the NCO. In fact, even though a second NCO could be synthesized into the design, the number of DSP blocks remaining would not be sufficient to fit 32 additional lock-ins, resulting in a suboptimal design.

Despite the fact that further optimizations can lead to even faster and more compact designs, it is important to emphasize that even the low-cost FPGA used in this work significantly enhances the acquisition timing. Also, such a technique can easily be synthesized in many devices with a larger number of embedded DSP blocks.

## 5. Conclusions

The optimization of custom readout electronics and processing algorithms is crucial to fully exploit innovative sensors, and it often represents a key element in advancing specific technologies. The frequency-multiplexed architecture reported in this work not only enables the simultaneous measurement of multiple sensors, significantly increasing acquisition speeds, but also allows for the easy scalability of the entire design.

To fully leverage the parallel nature of this sensor type, a matrix of custom, fully digital lock-in amplifiers was synthesized within a Cyclone^®^ V FPGA, resulting in a 32-fold increase in acquisition speed. The datapath design introduced in this paper exemplifies how the processing capabilities of FPGAs can significantly enhance the measurement and data analysis of various complex sensor architectures.

Despite sub-optimal sensing conditions and poor thermal conductivity, the measurements carried out on the bidimensional array of MTRs have demonstrated the feasibility of this readout approach and the good sensing performance in both the visible and sub-THz ranges.

Lastly, the readout system has the potential to be employed with an array of completely different devices with only minor adjustments, showcasing its versatility and adaptability.

## Figures and Tables

**Table 1:** Logic resources used for the main modules inside the Intel Cyclone^®^ V design.

	Logic Utilization	BRAM	DSP
	**[ALMs]**	**[M10K]**	
Design	17,012/29,080	137/446	101/150
Lock-in matrix	9193/29,080	3/446	96/150
Lock-in amplifier	145/29,080	0/446	3/150
2-Slow IIR filter	27/29,080	0/446	1/ 150
Network wrapper	3168/29,080	62/446	0/ 150

## Data Availability

The data are available upon request.
